# *In vitro *antioxidant activity of the prostatic secretory granules in rabbit semen after exposure to organic peroxides

**DOI:** 10.1186/1477-7827-8-16

**Published:** 2010-02-23

**Authors:** Evangelia Mourvaki, Raffaella Cardinali, Alessandro Dal Bosco, Cesare Castellini

**Affiliations:** 1 Department of Applied Biology, Section of Animal Science, University of Perugia, Borgo XX Giugno 74, 06100, Perugia, Italy

## Abstract

**Background:**

The prostate gland of rabbits produces numerous granules, which are specifically implicated in the inhibition of sperm capacitation during the first hours after mating. These granules are rich in vitamin E, but their role in the antioxidant protection of rabbit sperm has not been studied.

**Aim of study:**

The objectives of this study were to investigate whether the prostatic secretory granules (PSGs) could prevent sperm induced-lipid peroxidation and to verify a potential involvement of tocopherols from the PSGs to the sperm.

**Methods:**

Washed sperm samples taken from eighteen White New Zealand rabbits were either incubated with tert-butyl-hydroperoxide (t-BHP, an oxidative stressor) or with buffered Tyrode's medium for 1 hour. The same number of sperm samples that contained PSGs were subjected to the previously mentioned treatments and thiobarbituric acid reactive substances (TBARS), vitamin E compounds and the acrosome status were assessed.

**Results:**

The incubation of the sperm with t-BHP resulted in a noticeable production of TBARS (0.38 vs. 0.22 nmol/10(7) cells) and an associated decrease of alpha-tocopherol (alpha-T, 72.3 vs. 103.2 nmol/10(8) cells) with respect to the sperm samples containing PSGs. The sperm incubated with the PSGs had a higher amount of alpha-T compared to the control (292.2 vs. 251.0 nmol/10(8) cells). The acrosome status was not affected by the occurrence of the organic peroxide in the medium and the amount of capacitated sperm was lower when the PSGs were also present.

**Conclusions:**

Overall, these results suggest that the PSGs may represent a source of protection for rabbit sperm against in vitro oxidative stress by supplying the sperm with endogenous alpha-T. This mechanism could be in part involved in the inhibition of sperm capacitation by the granules.

## Background

The rabbit prostate produces and secretes a great number of membranous granules that range in size from 0.5-4 μm. These granules have several potential functions related to rabbit reproduction and fertility [1]. Similar multilamellar vesicles have also been isolated from human semen (namely, prostasomes) and from other animal species (namely, prostasome-like vesicles) [2-5]. The physiological roles of these prostate-derived granules have been amply debated and several hypotheses have been put forward. Among these roles, the promotion of spermatozoa motility [6], antibacterial activity [7], immunosuppression [8] and anticancer properties [9] have been related to the prostate granules in human men. In rabbits, we have recently found that these prostatic secretory granules (PSGs) are able to inhibit sperm capacitation, delaying the acrosome reaction, and that they promote sperm motility during the first hours after mating [10]. Both effects are fundamental requisites for successful egg fertilization in animals with an induced ovulation in which the spermatozoa have to wait about 16-18 h for the egg availability, such as in female rabbits [1,10].

The lipid composition of the PSGs is dissimilar to that of the sperm plasma membrane [11]. Cholesterol is the most abundant lipid and a considerable amount of vitamin E (vitE), approximately 38% of the entire semen content, is contained in such granules [12]. In particular, three of the eight naturally occurring components of vitE have been found in the PSGs in different proportions: α-, γ- and δ-tocopherol, representing 87.5%, 7% and 5.5% of the total PSG-related vitE, respectively. Although these compounds are known to possess substantial antioxidant activity [13], there is no direct experimental evidence concerning the potential role of the PSGs in the antioxidant protection of rabbit sperm via vitE, and whether this vitE-related biological function could be based on the of inhibition of sperm capacitation by the PSGs. It is well-known that although a certain level of reactive oxygen species (ROS) is required to drive sperm toward capacitation and the acrosome reaction [14], the high content of polyunsaturated fatty acids within the sperm plasma membrane renders these cells highly susceptible to oxidative damage, which may lead to sperm dysfunction and/or death [15].

The purpose of the current study was to investigate whether the PSGs could prevent the *in vitro *sperm lipid peroxidation induced by tert-butyl hydroperoxide (t-BHP), and to verify the potential role of the PSG-related tocopherols in this process. The effect of t-BHP on the acrosome status of the sperm in the presence or the absence of the PSGs was also evaluated.

## Methods

### Materials

Unless otherwise noted, all of the chemicals were analytical grade or high performance liquid chromatography (HPLC) grade and were purchased from the Sigma Chemical Company (St Louis, MO, USA). The protein dye reagent was obtained from BioRad Laboratories (Hercules, CA, USA).

### Animals, semen collection and fractionation

For the purposes of this study, eighteen fertile male New Zealand White rabbits (8 mo of age) were housed in individual cages with a photoperiod of 16 h light/day, at an intensity of 40 lux and temperatures ranging from 16 to 25°C [16]. All of the animals were fed ad libitum on a standard diet composed of dehydrated alfa-alfa meal (40%), soybean meal (18%), barley (30%), wheat bran (10%), mineral and vitamins (2%). The results of the diet chemical analysis (expressed as the % dry matter) were: crude protein (17.5%), crude fiber (15%), fat (2.5%), polysaccharides (58.3%) and ash (6.2%).

The semen collection was performed weekly by means of an artificial vagina kept at 37°C for a total of 2 wks (total semen samples, n = 36). After collection, the semen samples were immediately transferred to the laboratory of Animal Science, University of Perugia, for further processing. In detail, the volume and sperm concentration of the ejaculates were recorded by using a graduated tube and a Thoma-Zeiss cell counting chamber (BRAND, Wertheim, Germany) with a light microscope (Olympus CH-2, Olympus Optical Co. Ltd, Tokyo, Japan), respectively.

The percentage of the motile sperm cells and the curvilinear velocity of the sperm (VCL μm/sec) were evaluated using a Computer-Assisted Sperm Analyzer (CASA, model ISAS, Valencia, Spain) as previously described in [17].

To obtain enough material for the purposes of our study, semen samples were properly pooled.

The sperm were separated from the seminal plasma and from the PSGs using colloidal silica Percoll^® ^gradient-density centrifugation [18]. The pellets were re-suspended in a suitable volume of the extender with or without the lipid peroxidative stressor, as described below. The amount of PSGs in the ejaculated semen was determined by measuring their protein content according to the Bradford method after their reaction with Coomassie Brilliant Blue G-250 and using BSA as a standard [19]. The values were expressed as mg protein/ml of ejaculate.

### Incubation of sperm with tert-butyl hydroperoxide

To investigate whether the vitamin E content of the PSGs could play a preventative role against the lipid peroxidation of the sperm membrane, a fixed number of sperm (100 × 10^6 ^cells) were pre-incubated for 2 min with a given amount of pooled PSGs (0.25 ± 0.05 mg of protein). Following this, the entire specimen was incubated with 0.2 mM t-BHP at 37°C for 60 min in a Tyrode's Albumin Lactate Pyruvate buffer (TALP, [17]) or with only TALP (the control sperm). The same number of sperm were subjected to the aforementioned treatments (t-BHP or TALP) in the absence of the PSGs. Incubation was performed in an atmosphere containing 5% carbon dioxide. The experiment was repeated for ten times.

The concentration of the PSGs was established by taking into account their average concentration in the rabbit ejaculate (1.00 ± 0.32 mg protein/ml) [10]. Concerning the lipid peroxidative stressor, preliminary trials with various concentrations of t-BHP (50-500 μM) were tested to determine the optimal dosage (200 μM) in terms of cell mortality.

### HPLC determination of sperm vitamin E homologues

After incubation with t-BHP or with TALP and prior to the vitE determination, the sperm samples with and without the PSGs were subjected to a second Percoll^® ^gradient-density centrifugation. The pellets (sperm) were extracted twice with hexane after a double washing with PBS [12]. The chromatographic separation of the tocopherols was performed using a Jasco HPLC (PU-1520 equipped with a 7125 Rheodyne injector) system (JASCO Corporation, Tokyo, Japan) on a Beckman Ultrasphere-ODS column (5 μm particle size, 4.6 × 250 mm) (Beckman Coulter, Milan, Italy). The mobile phase consisted of methanol and acetonitrile (8:2) with ammonium acetate (100 mM). The flow rate of this phase was 1.2 ml/min. Concentrations of α-T, γ-T, and δ-T were quantified by fluorescence detection (Jasco, FP-1525) using the excitation and the emission wavelengths of 292 and 330 nm, respectively. Quantification was performed with reference to the external calibration curves prepared with increasing amounts of pure tocopherols in ethanol. The volume of the injection was 50 μL.

### TBARS determination

The extent of the sperm membrane lipid peroxidation induced by the incubation with the t-BHP in the presence or in the absence of the PSGs was assessed by measuring the malondialdehyde (MDA), along with other substances that are reactive to 2-thiobarbituric acid (TBA). MDA is an abundant secondary breakdown product of the peroxidized PUFA. To measure these compounds, washed sperm that were prepared as described above were incubated with a reaction solution at 95°C for 60 min and the resultant pink MDA-TBA adduct was quantified using a spectrophotometer (Hitachi 2000, Tokyo, Japan), set at 532 nm [20]. The molar extinction coefficient of the MDA was 1.56 × 10^5 ^M^-1^cm^-1^. The results are presented as nmol MDA/10^7 ^cells.

### Evaluation of the sperm viability and the spontaneous and induced acrosome reactions

The sperm viability was determined by using the Eosine Exclusion Test [21]. The acrosome statuses (the spontaneous acrosome reaction, the SAR and the induced acrosome reaction; IAR) of the spermatozoa were evaluated using fluorescein isothiocianate (FITC)-conjugated *Pisum sativum *agglutinin (PSA) staining with an epifluorescence microscope (Olympus CH-2, excitation filter 335-425 nm) [22]. A minimum of 200 cells/sample were scored according to the following staining patterns: (a) bright, homogeneous fluorescence in the anterior sperm head region, and (b) patchy fluorescence or staining limited to the equatorial segment, indicating an intact acrosome and a partially or totally reacted sperm, respectively. The percentage of the capacitated sperm was calculated by taking the difference between the sperm with IAR and those with SAR.

### Statistical analysis

The obtained data were analyzed using a linear regression model, taking into account the fixed effects of the presence of the PSGs and/or of the oxidative stressor. The results are presented as the mean and standard deviation [23]. Significant differences were evaluated using multiple t-tests (P < 0.05). Correlations between the studied variables were also evaluated by computing Pearson regression.

## Results

The semen characteristics are summarized in Table 1. The values obtained in this study were consistent with those reported for New Zealand White rabbits and confirm good semen quality [16,17].

**Table 1:** Rabbit semen characteristics (mean ± SEM)^x ^before incubation.

Semen	
Ejaculate volume, mL	0.60 ± 0.08
Sperm concentration, 10^8^/mL	4.06 ± 0.40
PSGs concentration, mg protein/mL	1.24 ± 0.05
Live cells, %	74.90 ± 5.00
Motile cells, %	72.40 ± 3.75
VCL^y^	201.33 ± 13.20

^x ^N = 36;

^y^VCL stands for curvilinear velocity of the sperm.

We found that the addition of 200 μM t-BHP to the sperm increased the cell mortality, as revealed by a 19% decrease in the sperm viability compared to the control sperm (table 2). This decrease was slightly lower for the sperm samples containing the PSGs. The rate of SAR and IAR and the number of capacitated sperm were not affected by the t-BHP, whereas the sperm samples containing the PSGs had a lower IAR and a lower capacitation, both in the presence and in the absence of the organic peroxide.

**Table 2:** Changes in live cells, spontaneous (SAR), induced (IAR) acrosome reaction, capacitated sperm, motile cells and VCL^y ^after treatment with prostatic secretory granules (PSGs) and/or tert-butyl-hydroperoxide (t-BHP) at 37°C for 60 min.

	Live cells %	SAR %	IAR %	Capacitated sperm %	Motile cells %	VCL μm/sec
Control sperm	75.0 b	20.4	68.4 b	48.0 b	60.8 b	195.0 b
Sperm+PSGs	80.8 b	16.3	50.1 a	33.9 a	61.7 b	205.1 b
t-BHP treated sperm	61.3 a	21.0	72.0 b	51.0 b	49.2 a	168.0 a
t-BHP treated sperm+PSGs	60.2 a	17.5	58.3 a	40.8 ab	56.7 b	197.2 b
Pooled SE	5.1	3.4	7.3	8.4	4.0	4.9

^y ^VCL stands for curvilinear velocity of the sperm;

a..b: Values with different letters on the same column are significantly different, P < 0.05.

Compared to the control samples, the number of motile cells and their speed decreased in the sperm that were treated with t-BHP (49.2% and 168 μm/sec, respectively) and this effect was less evident in the sperm samples with PSGs (56.7% and 197 μm/sec, respectively).

Regarding the lipid peroxidation, the presence of PSGs in the samples treated with the t-BHP reduced the susceptibility of the membrane lipids toward oxidation (Table 3). The incubation of the sperm with the organic peroxide resulted in a 2-fold increase in TBARS production compared to the control sperm.

**Table 3:** Sperm TBARS^y ^(nmol MDA/10^7 ^sperms) and tocopherol (T) levels (nmol/10^8 ^sperms) after treatment with prostatic secretory granules (PSGs) and/or tert-butyl-hydroperoxide (t-BHP) at 37°C for 60 min.

	TBARS	alpha-T	gamma-T	delta-T	Total - Tocopherols
Control sperm	0.19 a	251.0 c	31.5	1.7	284.2 c
Sperm+PSGs	0.15 a	292.2 d	15.4	0.3	307.9 c
t-BHP treated sperm	0.38 b	72.3 a	n.d.	n.d.	72.3 a
t-BHP treated sperm+PSGs	0.22 a	103.2 b	n.d.	n.d.	103.2 b
Pooled SE	0.04	7.2	0.8	0.3	30.9

^y ^TBARS stands for thiobarbituric acid reactive substances;

a..d: Values with different letters on the same column are significantly different, P < 0.05;

n.d. stands for non detectable.

The sperm vitE concentration was also affected by the occurrence of the PSGs during the induction of lipid peroxidation that occurred naturally or that was induced chemically. We found that the incubation of sperm with t-BHP for 60 min reduced their total tocopherol content by 75% (284.2 nmol/10^8 ^cells *vs *72.3 nmol/10^8 ^cells, P < 0.05), whereas a minor reduction (66%) was observed when the PSGs were present in the solution (307.9 nmol/10^8 ^cells *vs*. 103.2 nmol/10^8 ^cells, P < 0.05).

Among the tocol-derivatives, only the α-T increased by 16% (P = 0.05) after the incubation of the sperm with the PSGs, while the γ-T and the δ-T were dramatically reduced by 80 and 51% (P < 0.01), respectively. The γ-T and the δ-T tocopherols were both undetectable after the incubation of the sperm with t-BHP.

Lastly, there was a significant negative correlation between the TBARS levels, the number of motile cells, the VCL and the α-T (Table 4). The tocopherol was also correlated with the number of motile cells and their speed (r = 0.72 and 0.78, respectively). The IAR was positively correlated with the amount of capacitated sperm and with the TBARS, whereas the SAR was negatively correlated with the number of motile sperm cells and their speed. No correlations were found between the other parameters tested.

**Table 4:** Pearson correlations coefficient.

	Live cells	SAR	IAR	Capacitated sperm	TBARS	VCL	Motile cells
**SAR^x^**	-0.26						
**IAR^y^**	0.17	0.15					
**Capacitated sperm**	0.07	-0.06	0.91*				
**TBARS^z^**	-0.18	0.33	0.52*	0.56*			
**VCL^j^**	0.45	-0.64*	-0.35	-0.22	-0.62*		
**Motile cells**	0.74*	-0.52*	-0.19	-0.22	-0.65*	0.83*	
**α-tocopherol**	0.46	-0.30	-0.33	-0.34	-0.76*	0.72*	0.78*

^x ^SAR stands for spontaneous acrosome reaction;

^y ^IAR stands for induced acrosome reaction;

^z ^TBARS stands for thiobarbituric acid reactive substances;

^j ^VCL stands for curvilinear velocity of the sperm;

* P ≤ 0.05.

## Discussion

The membranes of rabbit sperm are very rich in long-chain PUFAs, which are vulnerable to free radicals, mainly hydroperoxide, because of their large number of double bonds. For this reason rabbit sperm may require a substantial amount of antioxidant protection [24]. In addition to the seminal plasma, we have recently found that the prostate-secreted granules that are naturally occurring in the ejaculated semen of rabbits contain considerable amounts of vitE (about 30 ng/mg protein: 88% α-T, 5% γ-T and 7% δ-T, respectively [12]), which is a powerful exogenous antioxidant against lipoperoxides. Accordingly, we have hypothesized that PSGs play a role in the protection of rabbit sperm from oxidative stress. To verify this hypothesis we evaluated the changes in the vitE content and in the TBARS levels of sperm incubated with or without t-BHP both in the presence and in the absence of PSGs.

The Tert-BHP is an organic peroxide that is used as a model for oxidative stress in cell culture experiments. This compound decomposes into other alkoxyl and peroxyl radicals in a reaction aided by metal ions that can generate ROS, including H_2_O_2_. The t-BHP induces radicals that accelerate the lipid peroxidation, cell toxicity through DNA damage and depletes the cell glutathione and protein thiols, resulting in general cell damage and apoptosis [25-27]. In our study, 200 μM of t-BHP significantly reduced the numbers of live and motile cells, and this was likely due to the aforementioned mechanisms, although in this paper only the lipid peroxidation and the vitE were studied. Indeed, a short time of exposure of the sperm to the t-BHP resulted in a significant increase in the TBARS (the late stage products of lipid peroxidation). The TBARS were negatively correlated with the motility rate of the sperm and the sperm α-T, which specifically scavenges peroxyl and alkoxyl radicals, breaking down the lipid peroxidation chain propagation. An irreversible loss of sperm motility due to lipid peroxides has also been reported by other researchers [15].

Regarding the role of the PSGs in oxidative stress, our data demonstrated for the first time that sperm samples free of PSGs are more susceptible to lipid peroxidation and lose a great part of their vitE content, compared to samples with PSGs. This finding suggests a direct involvement of PSGs in the antioxidant protection of sperm probably via their endogenous vitE content. In fact, sperm samples incubated with an appropriate amount of PSGs had a greater amount of α-T with respect to control sperm, further corroborating this assumption and giving a direct experimental evidence of the transfer of this antioxidant from the PSGs to the sperm. The additional α-T donated by the PSGs might have also accounted for the minor production of the TBARS after the incubation of sperm with the oxidative stress inducer in the presence of PSGs. However, the involvement of other compounds with antioxidant activity, such as glutathione, protein thiols or enzymes, cannot be excluded and their presence in the PSGs remains to be clarified.

Sperm are lacking the mechanisms for the synthesis of their own tocopherols and get these products from the surrounding medium through molecular mechanisms that are not fully understood. Vitamin E shares similar biochemical pathways with cholesterol regarding metabolism and transfer [28]. Several studies emphasize the involvement of albumin in cholesterol transfer from the seminal plasma into the sperm [29]. This protein, which is naturally occurring in the rabbit seminal plasma, was also present in the incubation medium, making the transfer of vitE from the PSGs to the sperm feasible. However, the interaction of the PSGs with the spermatozoa is a more complex process that depends on both the protein and the lipid composition of the PSGs [30,31]. Hence, further studies are required to elucidate the molecular mechanisms implicated in the vitE trafficking from the PSGs to the sperm.

Regarding the role of the other vitE homologues, we found that their concentration dramatically decreased, especially after the induction of oxidative stress, making their quantification unreliable. Moreover, there was no transfer of these tocols from the PSGs to the spermatozoa, apparently excluding their involvement in the antioxidant protection of the sperm by PSGs. Gamma- and δ-T are less hydrophobic than α-T, and therefore are believed to interact with the sperm membrane components more loosely, which may explain their low concentration levels in these cells [12]. In addition, their bioavailability and accumulation in tissues are strictly dependent on the degree of saturation of specific α-T binding proteins [28]. These considerations, taken together, may partially explain the lack of transfer of γ-T and δ-T from the PSGs to the sperm. On the other hand, the non-α-T isoforms are known to possess a greater *in vitro *antioxidant activity than α-T [32] and are able to enhance their inhibitory effect on lipid peroxidation when the act in synergy with α-T [33]. Based on these considerations, it could be hypothesized that γ-T and δ-T might have been preferentially used by the sperm and/or the PSGs against free radicals that are produced either spontaneously or artificially. To better understand the fate of these minor vitE compounds, the incubation of the sperm with pure standards under oxidative stress conditions is under investigation in our laboratories.

The acrosome status of the sperm was not affected by the organic peroxide, suggesting a minor involvement of the acrosome in the process of induced lipid peroxidation. These data are in accordance with the findings of Brouwers and Gadella 2003 [34] who used the same oxidative stresses inducer and the fluorescent probe C11-BODIPY^581/591 ^as lipid peroxidation index. These authors found an increase of lipid peroxidation in the mid-piece mitochondria of bovine spermatozoa treated with t-BHP, but not in the head, suggesting the presence of a more effective antioxidant system in the head of the sperm cell that is probably due to its precious content (the paternal DNA). A higher susceptibility of the sperm tail to free radicals explains the lower motility and slower speed of sperm challenged with the lipid-soluble ROS inducer.

Opposite to t-BHP, the presence of PSGs in the incubation medium reduced the rate of IAR, and was responsible for the lower number of capacitated sperm in the samples that were treated with t-BHP as well as those that were not. These findings, taken together, suggest that other molecules, such as cholesterol [3], might have been implicated in the inhibition of the sperm acrosome reaction without excluding the involvement of antioxidants in this process. In this respect, it is well known that a certain amount of ROS are produced by rabbit sperm during capacitation [35,36] and that this concentration should be modulated by the antioxidant system in order to protect the sperm cells from oxidative stress damage [37].

In conclusion, our findings suggest that PSGs may represent an additional source of protection to the rabbit sperm from oxidative stress, by supplying part of their endogenous α-T. Accordingly, the PSGs should be considered as important components in the process of fertilization, both for their attributes as modulators of sperm capacitation and the acrosome reaction and/or for their ability to preserve the sperm membrane from oxidative degradation while sperm remains in the female rabbit reproductive tract for an extended time. The role of the seminal plasma constituents in both of these biological processes is under investigation in our laboratories. Whether vitE is present in human prostasomes or if this compound is implicated in the antioxidant activity of these vesicles are some of the arising issues that future studies on human reproduction should address.

## Competing interests

The authors declare that they have no competing interests.

## Authors' contributions

EM, the author responsible for the study conception and design, performing HPLC analyses, interpretation of data and for manuscript drafting; RC, the author responsible for performing CASA analyses and acrosome status assessment by microscopy; ADB, the author responsible for animal care and manuscript sequence alignment; CC, the author responsible for the study conception, performing statistical analyses, interpretation of data and for manuscript drafting.

All authors read and approved the final manuscript.
